# Non-coding RNAs as novel biomarkers and therapeutic targets in breast cancer

**DOI:** 10.3389/or.2025.1621144

**Published:** 2025-08-29

**Authors:** Veronica Barbi, Sara De Martino, Aurora Aiello, Michela Gottardi Zamperla, Sara Negri, Luca Cis, Valeria Pecci, Simona Nanni, Antonella Farsetti, Fabio Martelli, Carlo Gaetano, Sandra Atlante

**Affiliations:** ^1^ Laboratory of Epigenetics, Istituti Clinici Scientifici Maugeri IRCCS, Pavia, Italy; ^2^ Department of Translational Medicine and Surgery, Università Cattolica del Sacro Cuore, Rome, Italy; ^3^ Institute for Systems Analysis and Computer Science, National Research Council (CNR) – IASI, Rome, Italy; ^4^ Fondazione “Policlinico Universitario A. Gemelli IRCCS”, Rome, Italy; ^5^ Molecular Cardiology Laboratory, IRCCS Policlinico San Donato, Milan, Italy

**Keywords:** breast cancer, epigenetics, non-coding RNAs, long non-coding RNAs (lncRNAs), circular RNAs (circRNAs), small non-coding RNAs (sncRNAs), hormone therapy

## Abstract

Breast cancer (BC) remains a leading cause of cancer-related morbidity and mortality worldwide. Its marked heterogeneity - encompassing molecular subtypes, histological characteristics, and variable therapeutic responses - continues to pose persistent clinical challenges Although advances in surgery, hormone therapy, chemotherapy, and targeted therapies have significantly improved patient outcomes, issues such as therapeutic resistance and disease relapse are still common, underscoring the need for novel molecular targets. Within this context, non-coding RNAs (ncRNAs) have emerged as pivotal regulators of breast cancer biology and hold promise as diagnostics and therapeutic agents. These non-protein-coding RNA molecules include diverse subclasses, such as long non-coding RNAs (lncRNAs), circular RNAs (circRNAs), and small non-coding RNAs (sncRNAs), each characterized by distinct structural features and biological functions. Mounting evidence implicates ncRNAs in key oncogenic processes - such as tumor initiation, progression, metastasis, immune evasion, and treatment resistance - often in a subtype-specific manner. Importantly, ncRNA expression profiles differ significantly across BC subtypes, and their stability in body fluids underscores their potential utility in liquid biopsy-based diagnostics. This review provides an integrated overview of the multifaceted roles of ncRNAs in BC, emphasizing their mechanisms of action, contributions to tumor heterogeneity, and translational potential as both biomarkers and therapeutic targets. Understanding ncRNAs complexity and context-specific functions may pave the way toward more precise, personalized interventions for BC patients.

## 1 Introduction

Breast cancer (BC) is one of the most common malignancies worldwide, responsible for 670,000 deaths reported globally and 2.3 million diagnoses. This tumor may interest both women and men, but the incidence is massively tilted for the female gender, representing 99% of the whole cases, against 0.5%–1% of the male group. Breast cancer is a type of tumor with different outcomes and presentations; it is a very heterogeneous disease in which both environmental and genetic factors are involved (World Health Organization - www.who.int/news-room/fact-sheets/detail/breast-cancer). Human breasts are paired mammary glands that develop in females during puberty under the influence of various pubertal hormones; it has an inner structure made of epithelial components consisting of lobules and ducts (distributed throughout the fibrous and the adipose tissues), leading out to the nipple (1).

**FIGURE 1:**
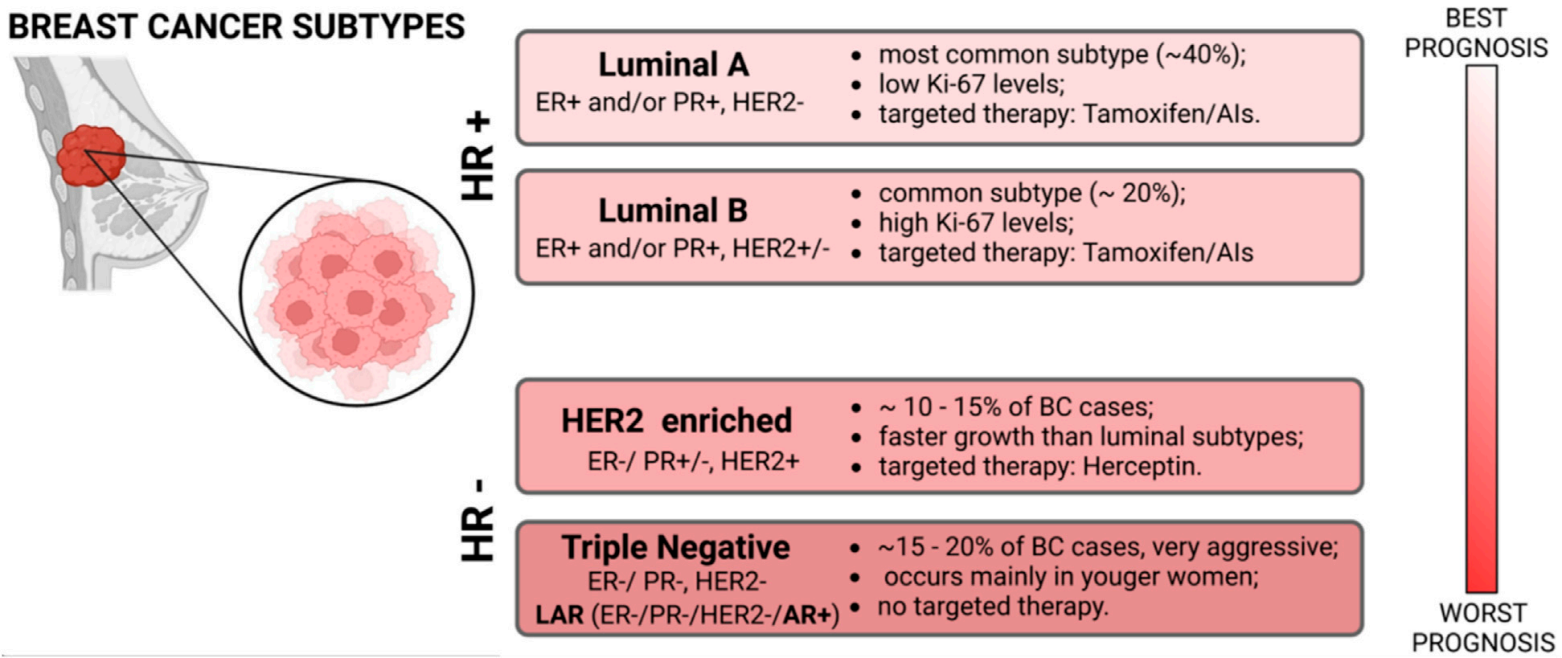
Scheme of the main molecular classification of Breast Cancer subtypes with their principal markers, histological grade, therapeutic approaches, and prognosis.

Most breast cancers are adenocarcinomas, and they can be invasive or non-invasive, according to their tendency to be circumscribed in the lobules and/or in the ducts (*i.e*., lobular carcinoma *in situ* and ductal carcinoma *in situ*) or to metastasize and “invade” other organs or tissues *(i.e.*, Paget’s disease, Triple Negative Breast Cancer (TNBC), medullary carcinoma, inflammatory breast cancer, mucinous carcinoma, tubular carcinoma, phyllodes tumor and infiltrating lobular/ductal carcinoma) (2–10). The possibility of developing breast cancer can be increased by various genetic or non-genetic factors, like the presence of mutations in breast cancer susceptibility one and two genes (*BRCA1* and *BRCA2*), smoke, obesity, or prolonged exposure to estrogen and progesterone hormones (11–13). Based on molecular and histological evidence, breast cancer comprises several histological and biological/molecular subtypes with distinct behaviors and responses to therapy: Luminal A, Luminal B, HER2 positive, and TNBC (Figure 1). Luminal A breast cancers are positive for the estrogen receptor (ER) and/or for the progesterone receptor (PR) but not for the Human Epidermal Growth Factor Receptor 2 (HER2); luminal B are ER+ and/or PR+ and HER2^+/−^, with a general higher proliferation rate; HER2 breast cancers are only HER2+, while TNBCs are ER-, PR- and HER2-. This last subtype represents the most aggressive form of the tumor, a feature determined by its ability to metastasize and by the lack of targeted treatment strategies. In fact, according to the molecular characteristics of each BC type, different approaches are adopted to reduce/suppress the tumor (14).

In luminal-like breast cancers, the standard procedure is represented by an endocrine therapy (15–17), while in HER2+ BCs an anti-HER2 humanized monoclonal antibody currently represents the most effective treatment (18). These compounds can be used alone or in combination with other procedures like surgery, radiotherapy, or chemotherapy, but a consistent portion of patients develop a resistance to the deputed drug (15). For triple-negative breast cancers there is no standardized treatment regimen and chemotherapy still represents the primary systemic treatment, but the efficacy of conventional post-operative adjuvant chemo-radiotherapy is poor (19). One exception is represented by a specific TNBC subtype named “LAR” (Luminal AR+) characterized by an enrichment in androgen response, fatty acid metabolism, and oxidative phosphorylation.

Interestingly, the LAR-subtype is closely related to the L2 (ER + luminal BC) hormone-responsive cells, and its cell lines were uniquely sensitive to the AR antagonist Bicalutamide; therefore, the next-generation AR antagonist Enzalutamide is currently being evaluated in AR-positive (AR+) TNBC in combination with Paclitaxel (20).

**FIGURE 2:**
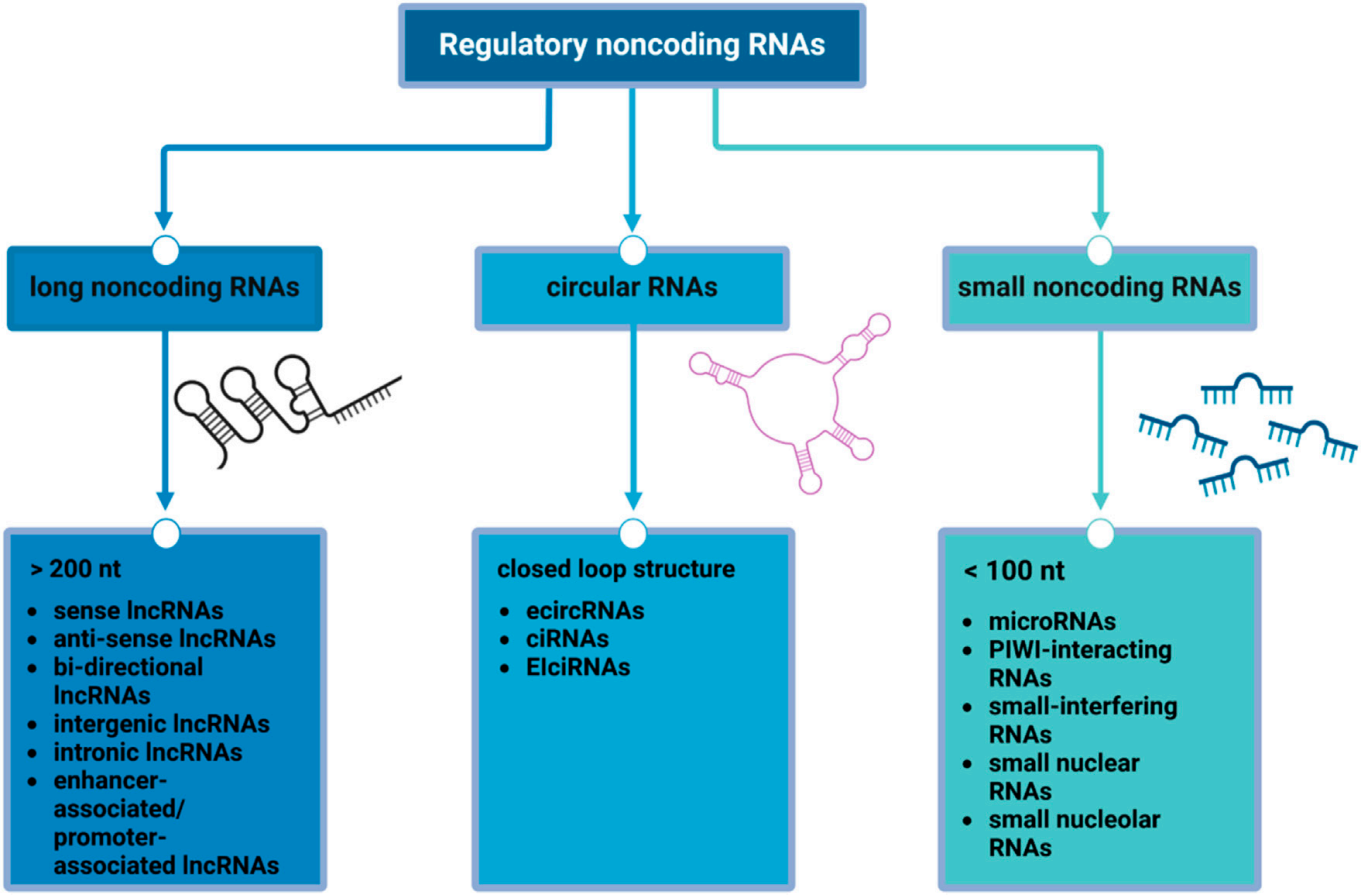
Regulatory non-coding RNAs. The scheme shows the main classes of non-coding RNAs according to their size and structure: long non-coding RNAs (lncRNAs), mainly involved in protein synthesis and epigenetic modifications and post-transcriptional processing; circular RNAs (circRNAs), acting as competing endogenous RNA, miRNA sponge, and regulators of alternative splicing and parental gene expression; small non-coding RNAs (sncRNAs), whose functions includes RNA silencing, RNA splicing, maturation and modifications, regulation of transposon activity and chromatin state and gametogenesis.

In this very heterogeneous and partially uncovered context, a pivotal role could be represented by epigenetics: understanding the intricate interplay of the epigenetic modifiers could be critical for unraveling the complexities of BC progression and developing targeted therapeutic interventions (21, 22). Non-coding RNAs are essential components of the complex epigenetic regulation machinery (Figure 2), and they play crucial roles in the post-transcriptional regulation of gene expression. Indeed, the dysregulation of their functions can result in unfavorable outcomes across various disease pathways (23).

Non-coding RNAs (ncRNAs) are RNA sequences that do not translate into proteins and can be generally categorized into three main classes (24): long non-coding RNAs (lncRNAs), small non-coding RNAs (sncRNAs) and circular RNAs (circRNAs). CircRNAs are a class of endogenous non-coding RNA, characterized by their covalently closed-loop structures without a 5′cap or a 3′poly(A) tail (25), while sncRNAs and lncRNAs are regulatory RNAs that differ in size (26).

## 2 Selection criteria and methodology

This review article aims to highlight the roles of non-coding RNAs in a very heterogeneous disease such as breast cancer. Over the years, many therapeutic approaches have been established and employed for BC, although they are inadequate or outdone in severe conditions. Given the avenue of new technologies and the growing body of research showing non-coding RNA involvement in BC tumorigenesis, progression, and invasion, this review aims to draw researchers’ and clinicians’ attention to the ncRNA’s potential use as biomarkers and therapeutic targets, examining their mechanisms and regulatory functions. We curated relevant studies primarily through a PubMed search using keywords tailored to each section of this manuscript, for example, “Epigenetics and Breast Cancer”, “non-coding RNAs in cancer”, “small non-coding RNAs and Breast Cancer”, “long non-coding RNAs and Breast Cancer” and “circRNAs and Breast Cancer”, deepening the research by focusing on peer-reviewed articles published in English, with a preference for those published during the last decade (2015–2025). Additional references were included based on cross-referencing and input from expert contributors to ensure thematic completeness and scientific accuracy.

We carefully evaluated the information extracted from these studies to ensure an accurate representation of the original research findings. The articles mentioned in this review were chosen prioritizing our purpose to give the reader an overview of every non-coding element in relationship with BC by following the same path: biogenesis, general biological role, eventual implication in tumorigenesis, invasiveness and migration and drug resistance, with a final focus on the consequent possible application as biomarkers or target for new advanced treatment. While every effort was made to include the most pertinent evidence, due to the volume of research in this area and the limitations inherent to a narrative format, we could not include every relevant article, and we apologize to the authors whose work could not be incorporated into this review.

This manuscript offers an overview of current knowledge in this field; despite the richness of the literature available, our work emerges as a comprehensive and didactic paper of particular interest for those who approach to the field for the first time and need a brief overlook to the theme. At the same time, the constant progress of the research highlights the need for adjourned reports of the state of the art.

## 3 Long non-coding RNAs

Long non-coding RNAs (lncRNAs) are transcripts longer than 200 nucleotides that are not translated into proteins and exhibit limited evolutionary conservation (27). Classification of lncRNAs is often based on their genomic positioning relative to protein-coding genes. Sense lncRNAs are transcribed from the strand as adjacent/overlapping protein-coding genes, while anti-sense lncRNAs are generated from the opposite strand. Bidirectional lncRNAs originate from the protein-coding gene’s promoter but are transcribed in the opposite direction. Intergenic lncRNAs are generated from regions between protein-coding sequences, and intronic lncRNAs are transcribed within the introns of coding genes (28). In addition, lncRNAs situated between two encoding protein genes can be classified into two main groups: enhancer-associated (elncRNA), which often regulate the expression of nearby genes on the same chromosome, and promoter-associated lncRNAs, which regulate chromosomal status and epigenetic inheritance (29–32).

**FIGURE 3:**
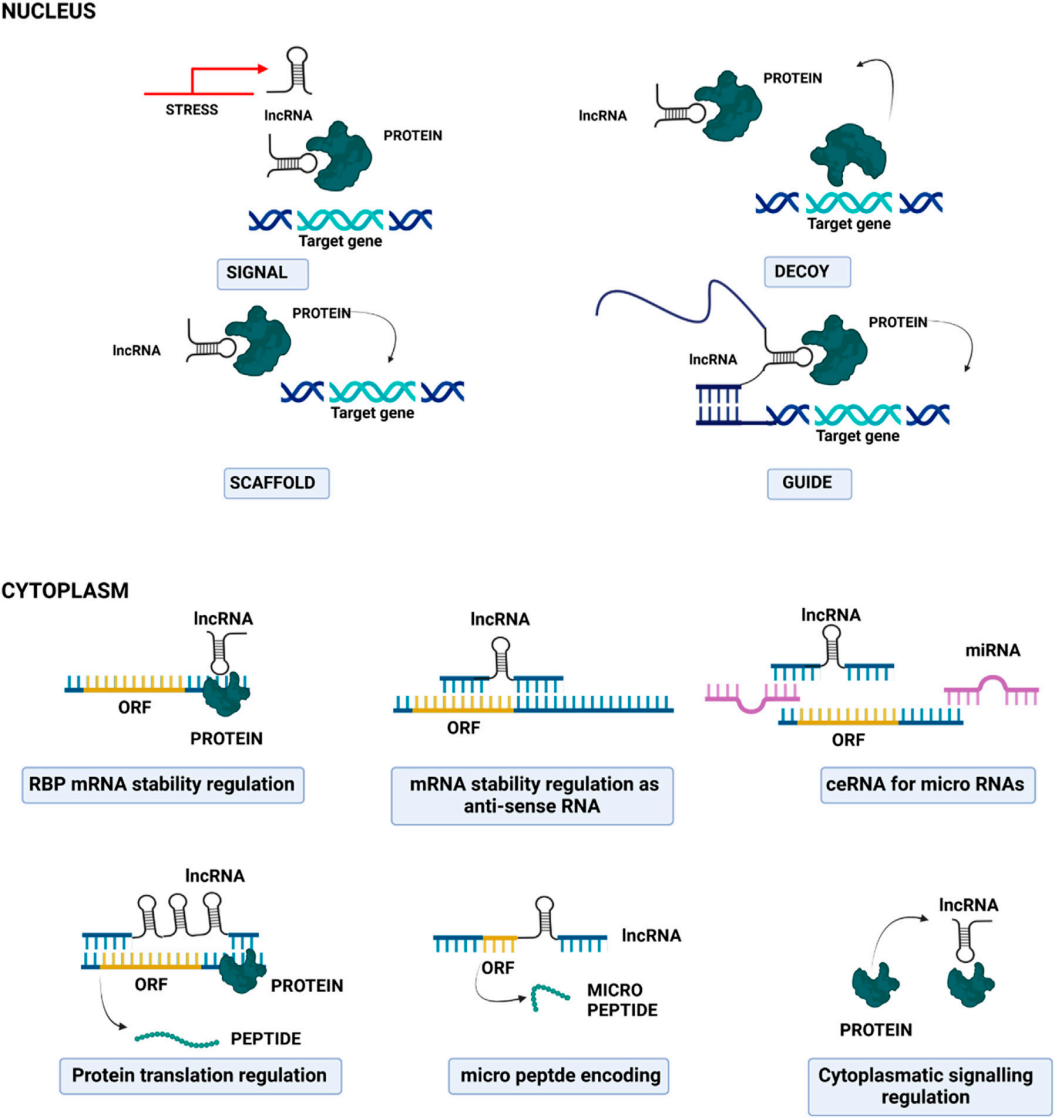
Cartoon depicting the principal molecular mechanisms of action of long non-coding RNAs in the nucleus (upper part) and in the cytoplasm (lower part).

The functional roles of lncRNAs are closely linked to their subcellular localization (Figure 3): in the nucleus, they participate in the modulation of epigenetic regulators and influence transcriptional programs through chromatin remodelling/interactions, and through the spatial organization of the nuclear compartment *via* scaffolding (33). Within the chromatin, lncRNAs can act as molecular scaffolds or guides for proteins, facilitating or inhibiting their recruitment and activity at specific genomic loci (34). In the cytoplasm, lncRNAs can regulate mRNA post-transcriptional processes, affecting mRNA stability and translation and modulating signaling pathways. Importantly, in the cytoplasm, they also act as miRNA “sponges”: they can bind miRNAs, thus reducing their availability to interact with target mRNAs, thereby indirectly regulating gene expression (35).

Furthermore, lncRNAs can be also transported into mitochondria, where they are implicated in the regulation of mitochondrial metabolism, apoptosis, and in their crosstalk with the nuclei (36). LncRNAs can also be packaged into exosomes, which are then released into the extracellular environment; next, exosome-localized lncRNAs can reach recipient cells, where they contribute to epigenetic regulation, cell-type reprogramming, and genomic instability (37).

Due to their extensive gene regulatory capabilities, lncRNAs influence a wide range of physiological processes, including cell differentiation, growth, and responses to diverse stresses and stimuli. Moreover, they play key roles in the nervous, cardiovascular, hematopoietic and immune systems and their associated pathologies. The involvement of lncRNAs in oncogenesis, specifically in cancer initiation and progression, is increasingly recognized. LncRNA exert their effects on cancer cell proliferation and survival, often by modulating key oncogenic or tumor-suppressive transcription factors, such as p53 (38–40).

In breast cancer, a growing body of evidence highlights the aberrant expression of specific lncRNAs across different BC subtypes, with strong correlations with tumor initiation, progression, and clinical outcomes (Table 1). Furthermore, lncRNAs are particularly attractive as therapeutic targets due to several advantageous properties: high tissue-specificity, regulation of specific elements of key cellular networks, limited toxic effects associated with their targeting, the often fast-turnover and their low expression levels, which could facilitate quicker effects with lower doses (41–43).

**TABLE 1:** Scheme of long non-coding RNAs implications in Breast Cancer types.

Long non-coding RNAs (lncRNAs)		
Regulation	lncRNAs	BC type
Breast cancer proliferation capacity/regulation of cancer stem cells (CSCs)	*LINC011333* (*KLF4* induction) (44); *LINC00511* (*miR-185-3p* sponge) (46); *LINC00617* (hnRNP-K recruitment) (47); *HOTAIR* (*miR-7* expression inhibition) (49); *EPB41L4A-AS2* (TSLNRs - cell apoptosis induction; H3K27 acetylation and EMT promotion) (48)	HR +, HER2 +, TNBC
Breast cancer metastasis	*MALAT1* (*miR-3064-5p* sponge; p53 inhibition; *MYC* regulation; Wnt signaling regulation) (51–56); *NBAT1* (H3K27me3 level reduction and Wnt signaling modulation; *EXH2* suppression) (57); *HOTAIR* (PRC2 recruitment) (58)	TNBC, HER2 +
Drug resistance	*H19* (Tamoxifen-resistance; *Beclin1* methylation downregulation and autophagy induction) (59); *AFAP1-AS1* (Trastuzumab-resistance; *ERBB2* translation promotion) (60)	HR +, HER2 +
Chemotherapy resistance	*BORG* (Doxorubicin/Adriamycin resistance; NF-kB signaling pathway activation) (61); *LINC00668* (Doxorubicin resistance, SND1 targeting) (61)	TNBC
Immune response	Downregulation of *XIST* (*C/EBPa* and *KLF6* expression inhibition) (62)	HR +, HER2 +, TNBC

LncRNAs can intervene in the regulation of breast cancer stem cells (BCSCs) related pathways; examples are the lncRNA *LINC01133* (which induces Kruppel-like factor 4 (*KLF4*) gene) (44, 45) and the long intergenic non-coding RNA 00511 (*LINC00511*) which functions as a *miR-185-3p* “sponge”, indirectly activating (*via* the E2F1 protein targeting) the transcription of Nanog, a promoter of regeneration and prolonged proliferative potential of stem-like cancer cells, able to mediate oncogenic reprogramming. Thus, the *LINC00511/miR-185-3p/E2F1/Nanog* axis may also have therapeutic potential, by regulating breast cancer stemness and tumorigenesis (46). Another important example is represented by *LINC00617*, which can also impact on the BCSCs self-renewal capacity through the activation of *SOX2* transcription mediated by hnRNP-K recruiting (47). Several long non-coding RNAs can also regulate breast cancer stem cells through epigenetic modifications, like the repression of the tumor suppressor long non-coding RNA (TSLNRs) *EPB41L4A-AS2* through the enrichment of H3K27me3 (48).

A long non-coding RNA that actively intervenes in the development and maintenance of BC is HOX transcript antisense RNA (*HOTAIR*) that inhibits *miR-7* expression, leading to increased *SETDB1* expression in BCSCs, inducing the epithelial-mesenchymal transition (EMT).

*HOTAIR* can additionally foster H3K27 acetylation and E-cadherin promoter methylation, this mechanism inhibits E-cadherin production furtherly promoting EMT (49).

Non-coding RNAs are also implied in breast cancer metastatic progression; as depicted in Figure 3, they can act by different mechanisms, such as the degradation or silencing of specific mRNAs, the target of enzymes and microprocessor subunits involved in miRNA biogenesis, and the sponging of miRNAs, thus altering the expression of several genes and modulating different cell signaling pathways. Metastasis-associated lung adenocarcinoma transcript 1 (*MALAT1*) has been correlated with an increased tumor size and stage, and a consequent poor prognosis in human patients: it undergoes tight transcriptional control in tumor cells by several transcription factors, both positively and negatively (50). For example, hypoxia-inducible factor 1α (*HIF-1α*) upregulates *MALAT1* with the mediation of AMP-activated protein kinase (AMPK) (51); the induced lncRNA then acts as a miRNA sponge of *miR-3064-5p*, a mechanism that promotes tumor growth and migration in breast cancer cells (52).

Conversely, the depletion of *MALAT1* triggers the arrest of the cell cycle followed by a reduced cellular proliferation rate. It activates p53 – a tumor suppressor that participates in apoptosis and senescence processes–and its target genes (53).

According to several studies, this long non-coding could be essential in developing and metastasizing TNBC and HER2-positive BC because the presence of metastatic lymph nodes is correlated with *MALAT1* expression in breast cancer patients. In addition, *MYC* and its downstream immune regulatory genes (*CD47* and *PD-L1*) are related to metastasis and relapse in these subtypes of BCs and are positively regulated by *MALAT1* (54)*.* Huang and colleagues demonstrated that the knockdown of *MALAT1* in MCF-7 cells reduced *EGF* expression, suggesting that it might initiate angiogenesis in BC, through modification of *miR-145* (55). According to multiple lines of evidence, *MALAT1* is also implicated in regulating signaling pathways associated with cancer progression, such as the Wnt signalling (56), but it is still uncertain how it affects these pathways.

Another important example of the contribution of the lncRNAs in breast cancer metastasis formation is represented by neuroblastoma-associated transcript 1 (*NBAT1*) (57), and by the aforementioned *HOTAIR*. The former induces BC cells invasiveness by reducing H3K27me3 levels, while the latter induces migration and invasion by recruiting the polycomb repressive complex 2 (PRC2) which leads to the variation of H3K27 methylation levels and global gene expression alterations (58).

LncRNAs can often interfere in protein translation; it was in fact shown that abnormally expressed lncRNAs can be also related to multidrug resistance in breast cancer. In endocrine therapies, the resistance to Tamoxifen can be mediated by the induction of an autophagy mechanism; this mechanism can be triggered by long non-coding RNAs–such as *H19* – acting on key mediators of the process (59). In HER2+ BCs, an augmented expression of the lncRNA *AFAP1-AS1* can induce resistance to Trastuzumab by binding to AUF1, thus promoting *ERBB2* translation (60). Furthermore, several lncRNAs can also be involved positively or negatively in the resistance to Doxorubicin/Adriamycin: lncRNA *BORG*, for example, can activate the NF-kB signaling decreasing the genomic damage, whilst *LINC00668* targets staphylococcal nuclease domain-containing 1 (SND1) and improves the resistance to DOX (61).

An altered immune response in the tumor microenvironment can also markedly affect cancer occurrence and development. In this context, lncRNAs can regulate the function of immune cells impacting the antigen presentation ability of dendritic cells (DCs): for instance, the lncRNA *XIST* (62) down-modulation in M1-type macrophages (M1) leads to the transformation in anti-inflammatory M2 macrophage (M2) to promote tumor cell proliferation and migration. Recent studies have shown that lncRNAs can intervene in immunosuppression and may be a potential target for cancer immunotherapy, but the mechanism of tumor immune escape is highly complex and needs to be extensively investigated (62). Undoubtedly, they are promising predictive biomarkers and therapeutic targets for breast cancer immunotherapy, although further research is still required.

All these correlations make long non-coding RNAs good candidates as biomarkers for tumor diagnosis and prognosis and for predicting disease progression, but also as therapeutic targets in the shape of small molecule inhibitors, siRNAs, antisense oligonucleotides (ASOs), and CRISPR-Cas9. Vaidya and Collogues, for instance, studied the Differentiation Antagonizing Non-Coding RNA (*DANCR*), which is a non-coding RNA involved in the regulation of different oncogenic mechanisms and undruggable by conventional molecules; it was demonstrated that the delivery of siRNA against *DANCR* and its subsequent inhibition epigenetically represses the expression of cancer-driven pathways, such as Wnt signaling, EMT, and phosphorylation of several kinases: siDANCR-NP effectively inhibits migration and invasion of cancer cells *in vitro* and tumor growth *in vivo* (63). A small molecular inhibitor, AC1Q3QWB, has been implemented in breast cancer-rived xenografts, resulting in efficient disruption of PRC2 recruitment by *HOTAIR* without notable off-target effects (64).

On the other hand, these compounds are not easy to design, and although lncRNAs are opening a new door for clinical diagnosis and treatment of breast cancer, there are still many difficulties that must be faced and overcome.

## 4 Circular RNAs

**FIGURE 4:**
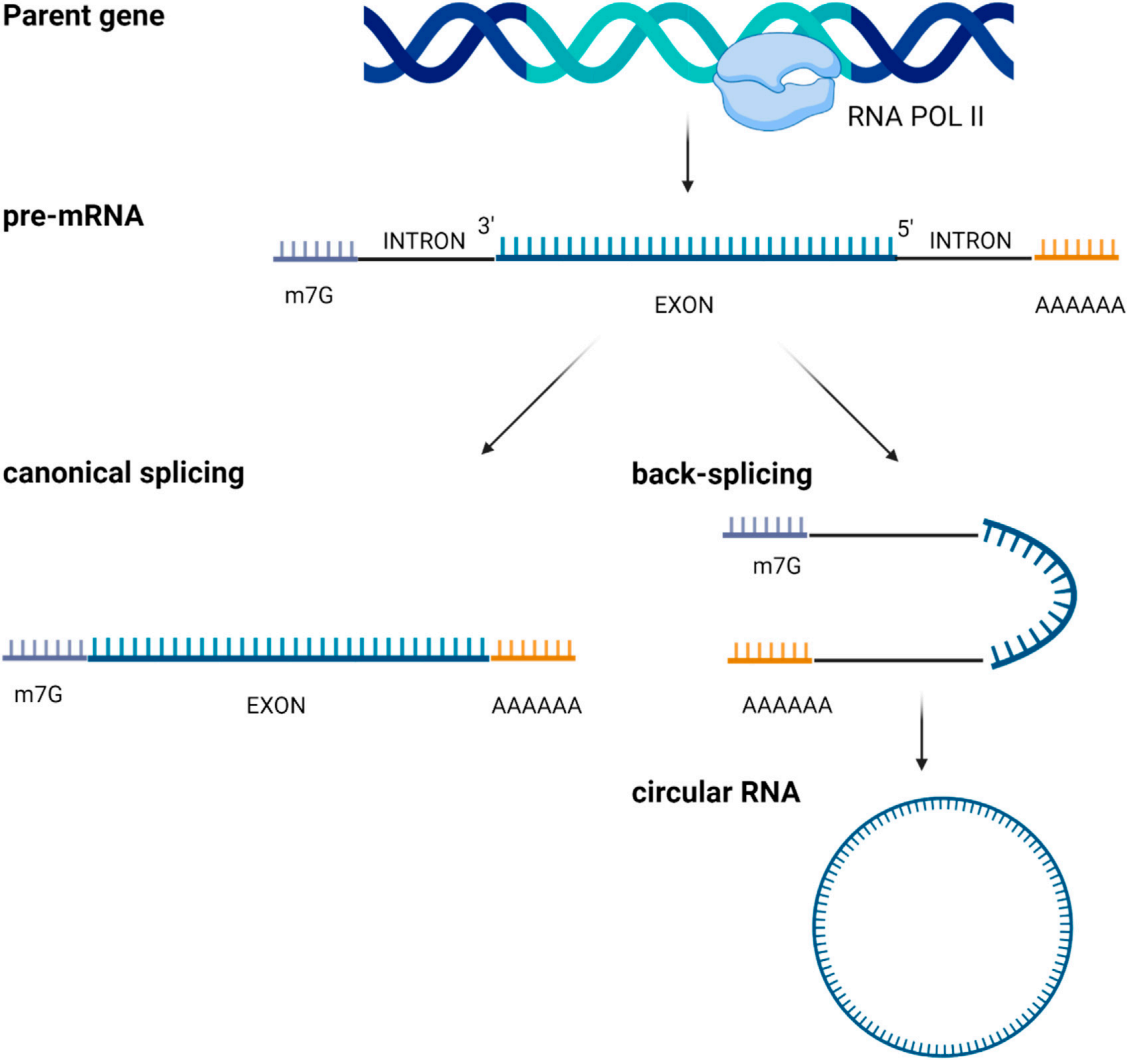
Scheme of circular RNAs biogenesis through mRNA back-splicing of exons.

Circular RNAs (circRNAs) represent a subclass of single-stranded RNAs derived from precursor mRNAs or lncRNAs and characterized by a covalently closed loop structure. CircRNAs are generated by a process known as “RNA back-splicing” in which the 3′-end of an exon is joined to the 5′-end of the same or an upstream exon, *via* a 3′, 5′-phosphodiester bond. This event creates a closed circular structure containing a characteristic back-splicing junction (Figure 4).

CircRNAs are broadly categorized based on their composition. Most of them are composed only by exonic sequences and are referred to as exonic circRNAs (ecircRNAs). Less frequently, circRNAs may be formed entirely from intronic regions (IcirRNAs) or may contain both exonic and intronic sequences (EIciRNAs). EcircRNAs are mostly localized in the cytoplasm, although the precise mechanism governing their nuclear export remains insufficiently understood (65); interestingly, some ecircRNAs are found within the nucleus where they increase the nuclear retention of specific proteins or recruit proteins to chromatin (66). Conversely, most intron-containing circRNAs are retained in the nucleus, where they may regulate their parental gene expression (67).

Recent investigations have extended our understanding of circRNA biology by identifying a subset of circRNAs localized in mitochondria, extending the complexity of the mitochondrial transcriptome (68–70). However, the presence and functional relevance of circRNAs in other organelles and subcellular compartments remain largely unexplored and warrant further study.

The unique structure of these RNAs makes them more resistant to exonucleases than their linear counterparts, providing them with a longer half-life; in fact, circRNAs are often stable and accumulate in most cell types, with an especially high abundance in neural tissues. These features make circRNAs attractive candidates as diagnostic biomarkers and therapeutic targets.

A growing body of research has documented the distinct expression profiles and functional significance of circRNAs in a range of pathological conditions, including cancer (70–72), cardiovascular disease (73), neurological disorder (74), and autoimmune disease (75). Despite these advancements, the mechanisms underlying the abnormal landscape of circRNAs and how circRNAs exert their physiopathological roles remain poorly understood. Table 2 summarizes key circular RNAs involved in the BC pathological processes.

**TABLE 2:** Scheme of circular RNA implications in Breast Cancer types.

Circular RNAs (circRNAs)		
Regulation	circRNAs	BC type
Breast cancer proliferation/tumorigenesis	Has_circRPPH_015 (oncogenic sponge) (77); circ-Amotl1 (66); circPVT1 (97)	HR+, HER2+, TNBC
Breast cancer progression/metastasis	Has_circRPPH_015 (oncogenic sponge) (77); circPSMA1 (81); circACTN4 (97); circSEPT9 (98); circEZH2 (84); circROBO1 (85)	HR+, HER2+, TNBC
Drug resistance	Has_circRPPH_015 (oncogenic sponge) (77); circCDYL2 (Trastuzumab resistance) (87); circRNA-CREIT (88), circUBE2D2 (92) (Doxorubicin resistance); circRNA-SFMBT2 (Tamoxifen resistance) (89)	HR+, HER2+, TNBC
Tumor suppression	circBMPR2 (99); circSMARCA5 (78)	HR+, HER2+, TNBC

Of note, one of the main features of circular RNAs is that they can act as miRNA “sponges,” or competitive inhibitors. By circRNAs interaction/sequestration, miRNAs are prevented from binding to their mRNA targets, inhibiting miRNA-mediated gene silencing and protecting target mRNAs from degradation (76). Some circRNAs can be classified as oncogenic sponges, as they facilitate multiple malignant behaviors, including tumor proliferation, distance metastatization, and drug resistance. An example is *Has_circRPPH_015*, which is upregulated in BC tissues. At the same time, its knockdown restrains aggressive behaviors of BC cell line MCF-7: in fact, this circRNA can bind to *miR-326* and negatively regulates ELK; on the contrary, an elevated expression of *miR-326* inhibits cell proliferation, colony formation, and cell invasion in this BC line (77).

CircRNAs bind often to transcription factors promoters, regulating their expression. At the same time, they can also function as scaffold in the modulation of protein-protein interactions, or even have translational potential.

CircRNAs participate both positively and negatively in breast cancer development and progress, acting as either oncogenes or tumor suppressors and their aberrant expression can be associated with tumoral cell proliferation, apoptosis, autophagy, invasion, migration, and treatment resistance.

A differential expression of circRNAs has been recently associated with diverse breast tumor status, drawing attention to the possibility to outline a circRNA “signature” in different tumor biopsies or cell lines. Cancer cells can in fact release these ncRNAs in urine, plasma, and saliva, opening the possibility of using circular RNAs as potential biomarkers in diagnosis and prognosis, as for the case of *circSMARCA5*, *Hsa_circ_0104824* that were shown to be decreased in BC patients’ blood compared to controls (78, 79). In other studies, *circPSMA1* appeared upregulated in serum/plasma of BC patients compared with those of healthy controls (80–82). More specifically, the overexpression of *circPSMA1* promoted TNBC cell proliferation, migration, and metastasis both *in vitro* and *in vivo* (81), together with another circRNA - *circ-Amotl1* - which is involved in tumorigenesis, enhancing the stability of c-MYC and the expression of its targets (66). *CircPVT1* can work through both ceRNA and protein scaffolding mechanisms: it sponges *miR-181a-2-3p* to modulate *ESR1* mRNA stability and downstream estrogen/ERα-target genes, while it represses type I IFNs and ISGs by binding MAVS to disturb RIGI–MAVS complex formation. This dual function contributes to ERα-positive BC development (83). CircRNAs are thus becoming more clinically relevant in breast cancer diagnosis, particularly for their early detection and stratification into different subtypes; which ameliorates the disease prognosis; however, they have been poorly explored in HER2-related BC subtypes, and more investigations are needed.

Beyond tumorigenesis, these non-coding RNAs also play a role in progression and metastasis: in a recent work, Peng and colleagues showed that an overexpression of *circEZH2* impacted on the vitality and the invasion of breast cancer cells, while its knockdown led to the opposite effects (84). The same molecular mechanism is furtherly used by *circROBO1*, another important actor in the migration and invasiveness of BC cancer cell, especially in liver metastasis (85).

Resistance to treatments represents still a challenging issue in breast cancer therapy and survival; in this scenario, a more personalized approach could represent a significant improvement and circRNAs might be promising predictive biomarkers, according to their ability to regulate BC cell sensibility to drugs/treatments (86). For example, the circular RNA *circCDYL2* confers Trastuzumab resistance in BC patients by stabilizing GRB7 and preventing its ubiquitination degradation; this enhances its interaction with FAK, which thus sustains the activities of downstream AKT and ERK1/2 (87). A recent work demonstrated that *circRNA-CREIT* is aberrantly downregulated in Doxorubicin-resistant TNBC cells: the RNA binding protein DHX9 is responsible for its reduction by interacting with the flanking inverted repeat Alu (IRAlu) sequences and inhibiting back-splicing. Mechanistically, *circRNA-CREIT* acts as a scaffold for proteins interaction, affecting the PKR/eIF2α signaling axis - related to stress granules (SGs) assembly - and the RACK1/MTK1 apoptosis signaling pathway. Further investigations revealed that a combination of the SG inhibitor ISRIB and Doxorubicin synergistically inhibits TNBC tumor growth. Besides, *circRNA-CREIT* could be packaged into exosomes and disseminate Doxorubicin sensitivity among TNBC cells (88).

In hormone therapies, Zheng and colleagues observed that *circRNA-SFMBT2* appeared to be directly related to cell proliferation and Tamoxifen resistance *in vitro* (89), whereas *circ_0025202* has been reported as a potential predictive biomarker of BC resistance to Tamoxifen (90), but more research is needed to confirm the data.

In the end, circular RNAs can be potential biomarkers also in chemotherapy resistance, as well as radiotherapy and immunotherapy resistance. The inhibition of *cirCDR1as*, for example, increases the sensitivity to 5-fluorouracil and Cisplatin of initially resistant BC cells, while an augmented expression of *circSMARCA5* improves the chemosensitivity to Cisplatin of BC cells and tumors (78). *CircKDM4C* is strongly associated with Doxorubicin resistance cells both *in vivo* and *in vitro* being a potential biomarker for a Doxorubicin response prediction (91). Similarly, *circUBE2D2* is involved in Doxorubicin resistance in TNBC cells, acting at the cellular level as a sponge of *miR-512-3p*, resulting in the upregulation of *CDCA3* expression (92). Several studies have also investigated the role of circRNAs in ADM-resistance (93) and the resistance to taxanes (94, 95). Lastly, a recent work by Li and colleagues reported that the circular HER2 RNA (*circHER2*) encodes for a novel protein–HER2-103 – that is expressed in a marked percentage of TNBC cases, with a worse overall prognosis than *circ-HER2*/HER2–103 negative patients; in their work, HER2-103 enhanced both homo and hetero dimerization of EGFR/HER3, AKT phosphorylation and malignant phenotypes.

Furthermore, Pertuzumab, an antibody employed in HER2+ tumor treatment, could represent a potential antagonist due to the congruence of the amino acid sequence of HER2-103 and HER2 CR1 domain. This antibody in fact decreased the *in vivo* tumorigenicity only of the triple-negative tumoral cells expressing the *circ-HER2*/HER2–103 (96). Altogether, these studies show that circRNAs may predict responsiveness to chemo-, radio-, immuno-, and hormone-therapies, and further clinical investigations are encouraged for validation.

## 5 Small non-coding RNAs

Small non-coding RNAs (sncRNAs) are a class of highly abundant ncRNAs that are typically <100 nucleotides (nt) long, transcribed from noncoding genomic regions with the ability to regulate various aspects of gene expression during normal animal physiology and development. sncRNAs control gene expression by regulating chromatin architecture, transcription, RNA splicing, editing, translation, and turnover. They are further divided into different subtypes: micro RNAs (miRNAs), PIWI-interacting RNAs (piRNAs), small-interfering RNAs (siRNAs), small nuclear RNAs (snRNAs) and small nucleolar RNAs (snoRNAs) (100).

### 5.1 MicroRNAs

MicroRNAs constitute a subgroup of abundant endogenous small noncoding RNAs made by single-stranded RNAs of approximately 19–24 nt length (26). Almost 2,500 putative miRNAs are currently identified in the human genome, but the number is increasing rapidly due to the development of high-throughput sequencing technologies. Approximately 50% of miRNAs are located in chromosomal regions prone to structural changes, making them crucial regulators of gene expression and promising candidates for biomarker development (101).

In general, micro RNAs target messenger RNAs that contain stretches of a complementary sequence to decrease their expression, although many miRNAs can also act on other non-coding RNAs (102); it is also known that one single miRNA can have more than one target and that one single gene can be modulated by more than one miRNA (103). A large body of works revealed the important role of miRNAs in many biological functions such as development, cell differentiation, embryogenesis, metabolism, organogenesis, and apoptosis (104). Furthermore, it has recently been proposed that circulating miRNAs could potentially contribute to intercellular communication and be introduced as targets of therapeutics for the treatment of different diseases (105).

MicroRNAs can also be localized extracellularly (such as in plasma/serum, urine, saliva, and seminal fluid), conserving more stability than cellular miRNAs (106–108). Some extracellular miRNAs are just products of cellular activities. However, many researches highlighted the importance of these miRNAs in different regulation processes and multiple studies have demonstrated that extracellular miRNAs can exert biological functions in recipient cells to regulate their activity, thereby acting as intercellular signaling molecules. *miR-105* is in fact expressed and secreted by metastatic breast cancer cells, in a potent regulator of migration through the target of ZO-1 (109, 110). MicroRNA expression patterns are frequently dysregulated in cancer, and great differences may be observed between normal and cancerous tissues and between localized and aggressive forms of cancer, depending on the type and stage of the disease (Table 3). It has been shown that certain microRNAs can induce oncogenesis, while others are involved in regulating gene targets associated with metastasis; they can either enhance or suppress the cancer phenotype by targeting tumor suppressor genes or oncogenes. Oncogenic miRNAs are often referred to as oncomiRs and are overexpressed in cancer cells, while tumor-suppressor miRNAs are usually downregulated, suggesting a significant role in cancer progression and representing potential targets for therapeutic intervention (23, 111).

**TABLE 3:** Scheme of miRNAs implications in Breast Cancer types.

Micro RNAs (miRNAs)		
Regulation	miRNAs	BC type
Breast cancer proliferation/tumorigenesis (oncomiRs)	miR-17∼92 cluster; miR-17; miR-18a; miR19a (123)	HR +, HER2 +, TNBC
Breast cancer metastasis	miR-105 (target ZO-1) (110); miR125b; miR-27a/b; miR-210; miR-30; miR-135-5p; miR-155 (118–121)	HER2 +, TNBC
Drug resistance	miR-221 (Tamoxifen-resistance) (117); miR-4728-3p (Lapatinib resistance) (119)	HR +, HER2 +
Chemotherapy resistance	miR-155 (124–126)	TNBC
Tumor suppression	miR-30 (cell division inhibition targeting cyclin D2); miR-99a (HOXA, mTOR, IGFBP1, FGFR3 inhibition) (113–116)	HR+, HER2+, TNBC
Anti-metastatic/anti-proliferative	miR-21; miR-10-b; miR-34a (116)	TNBC

In breast cancer, miRNA expression patterns also vary among the different subtypes. Luminal A and luminal B are very similar, but they differ in a more prominent dysregulation of subtype B compared to the A, which shows an abnormal regulation of 657 miRNAs against only 67 of the counterparts (112–116). Luminal A manifests a strong reduction in *miR-1290*, together with a downregulation of *miR-29a*, *miR-181a*, and *miR-652* and enrichment of *miR-30c-5p*, *miR-30b-5p*, and *miR-99a/let7c/miR-125b* cluster (113). Notably, in luminal A there is an evident presence of miRNAs associated with tumor suppression coherently with the low proliferation grade of this BCs subtype: *miR-30* for instance, inhibits cell division through cyclin D2 targeting, or *miR-99a* which reduces tumor growth by inhibiting proteins such as mTOR signaling (112). Conversant enrichment of *miR-182-5p*, *miR-200b-3p*, *miR-15b-3p*, *miR-149-5p*, *miR-193b-3p* and *miR-342-3p* defines, on the contrary, luminal B signature (112–116). In luminal-like breast cancers, microRNAs can also have a role in treatment responses: Tamoxifen resistance is an important issue in treating this neoplasia, reducing the success of therapy and resulting in either recurrence or metastatic or advanced-stage disease. It has been noticed that *miR-221* can provoke resistance to Tamoxifen by altering the cell cycle and evading apoptosis. It also regulates some signaling pathways like the Cip/Kip family (p21, p27, and p57), ERα, and phosphatase and PTEN. These regulations can lead to an increased proliferation and survival of BC cells and a decrease in apoptosis (117).

Numerous microRNAs are also associated with HER2+ breast cancer, in particular *miR-125b* (connected to metastasis and worst patient outcomes) is reported to be upregulated, while *miR-181d* and *miR-195-5p* are downregulated (118). *miR-4728-3p* is encoded within a *HER2* intron, and its mRNA targets include downstream targets of *HER2* signal transduction and the estrogen receptor alpha (*ESR1*). This microRNA is strongly related to HER2+ BC subtype and when its expression is particularly increased, the efficacy of *HER2* inhibitor Lapatinib is minimized (119).

Of note, microRNAs are expressed in a context-dependent manner, lying upon an evolving transcriptome, thus identifying changes in their landscape before and after eventual treatments could be helpful in the development of improved therapies, especially in cancers, when there is a shift in the abundance of relative target mRNAs during tumor progression (116).

In the end, TNBCs have also been seen in correlation with miRNA expression profile; in particular, *miR-27a/b*, *miR-210*, and *miR-30* are associated with worse survival and *miR-155* and *miR-493* are conversely associated with better outcomes (120, 121). Some micro RNAs associated with TNBCs are also reported to be associated with metabolic processes. For example, *miR-210* is involved in glucose uptake, lactate production, and extracellular acidification rate (120, 121). Generally, there is an increase in the expression *of miR-135b*, a non-coding RNA that regulates the expression of *ER*, *AR*, and hypoxia-inducible factor 1 alpha subunit inhibitor (*HIF1AN*), probably participating in the typical loss of hormone receptor of TNBCs. Several studies also highlighted the upregulation of *miR-135-5p*, which regulates migration processes in BC, with the functional differences among different subtypes arising from context-specific signaling networks (122).

Most of the triple-negative breast cancer molecular subtype data of miRNA associations is on BL1 and BL2, but it is very likely that there are specific correlations with other molecular subtypes even if they remain not completely clear yet. Basal-like triple-negative breast cancers manifest a signature of overexpression of the *miR-17∼92* (*iR-17*, *miR-18a*, *miR-19a*, *miR-20a*, *miR-19b-1*, and *miR-92a-1*) and *miR-106b-25* clusters; the proto-oncogene *cMYC* regulates the former to modulate the critical transcription factor E2F1, resulting in cancer proliferation. This miRNA cluster is often referred to as *oncomiR-1*, and it can also inhibit the inositol polyphosphate-4-phosphatase type II B (INPP4B) and associates with the BL1TNBC subtype. BL1 and BL2 show a difference in the expression of the *miR17∼92* cluster, *miR-17*, *miR-18a*, and *miR-19a*, which is lower in the latter (123).

MicroRNAs could also be perfect candidates for a new class of non-invasive biomarkers for diagnosis, prognosis, and therapeutic evaluation of cancer. Circulating miRNAs present in serum and plasma are highly stable and tissue-specific, as their collection in whole blood is undoubtedly a non-invasive and reproducible technique. Circulating levels of miRNAs are known to return to baseline levels after tumor removal, which justifies the potential usefulness of circulating miRNAs as biomarkers of cancer treatment efficacy. One of the most representative examples is *miR-155*, whose levels are significantly increased in breast cancers (likely contributing to cancer metastasis and chemotherapy resistance), and restored after therapy; while the mechanisms is not completely determined yet, these observations suggest that *miR-155* could represent a valid biomarker also for the tumoral stage identification (124–126). The high relevance of these small non-codings in breast cancer suggests that they have therapeutic potential that could be achieved *via* oncogenic miRNAs suppression/silencing or through the enrichment of tumor suppressive ones. In the first case, it is possible to eliminate the oncogenic miRNAs by delivering an oligomer complementary (referred to as antagomir), which binds to the mature miRNA, resulting in inhibition and degradation of the target. The counterpart is represented by an enrichment in tumor-suppressive miRNAs, reached through the delivery of miRNA mimics (double-stranded RNA sequences with the same sequence as the miRNA) in cells. In this context, there are three molecules primarily studied: *miR-21*, *miR-10-b*, and *miR-34a* that have shown a profound preclinical therapeutic potential, with both anti-metastatic and anti-proliferative properties (116).

Indeed, further analysis of the role of specific miRNAs and novel agents for manipulating tumor-specific miRNAs is required.

### 5.2 PIWI-interacting RNAs

PIWI-interacting RNAs (piRNAs) are a class of small non-coding RNAs with a length of generally 26–31 nt that interact with members of the PIWI family of proteins specifically expressed in germ cells to form a silencing complex named piRISC. PiRNAs originate from intergenic repetitive elements in the genome called piRNA clusters (approximately 186 in the whole human genome); in mammals, these clusters are dispersed within the chromosomes comparatively randomly, but synthetically preserved (127).

PIWI is widely expressed in various tumors, including seminomas, prostate, breast, gastrointestinal, ovarian, and endometrial cancer, and could act as an oncogene (12, 128). Since this evidence, researchers have started to assume a possible role of piRNAs in cancer and/or oncogenesis. In recent years, growing data supports the link between piRNAs and tumors: their abnormal expression is associated with various cancers and may play a pro-cancer or anti-cancer role in initiation, progression, and metastasis (Table 4). For example, an aberrant upregulation of *piR-651* has a crucial function in carcinogenesis in different types of cancers, like colon, lung, gastric and breast (129). In fact, this piRNA pathway plays a role in the balance between self-renewal and cell division and the perturbance of this symmetry may strongly impact tumor progression. *PiR-651* overexpression significantly promotes cell proliferation and migration of breast cancer cells by markedly reducing cell apoptosis and arrested cells in the G2/M phase by regulating the cell cycle.

**TABLE 4:** Scheme of PIWI-interacting RNAs implications in Breast Cancer types.

PIWI-interacting RNAs (piRNAs)		
Regulation	piRNAs	BC type
Tumorigenesis	*piR-651* (129–131); *piR932* and PIWIL2 protein (134); *piR-823* (136, 137)	HR+, HER2+, TNBC
Breast cancer proliferation/metastasis	*piR-651* (129–131); *piR-4987* (132); *piR-021285* (133); *piR932* and PIWIL2 protein (134)	HR +, HER2 +, TNBC
Drug resistance	*piR-651* (Tamoxifen-resistance) (129–131)	HR +
Tumor suppression	*piR-36712* (138); *piR-2158* (143); *piR-YBX1* (144, 146, 148, 149)	TNBC

Additionally, *piR-651* contributes to the methylation level modulation of *PTEN* promoter, and its consequent downregulation is also directly related to Tamoxifen resistance (130, 131); this highlights the potential role of this PIWI-interacting RNA as a potential diagnostic indicator and therapeutic target in the management of breast cancer (131).

BC promotion and progression has been found in association with several piRNAs, such as *piR-4987* – which is associated with lymph node positivity and poorer outcomes (132) – and *piRNA-021285*, which mediates the methylation of some related oncogenes in the tissues, representing a potential regulator of invasive BC (133). Recent studies also highlighted that the PIWIL2 protein works in combination with *piR-932* influencing the biological behavior of BCSCs through the methylation of Latexin (LXN) gene (134), coding for a tumor suppressor protein which reduces the risk of old stem cells transforming into cancer stem cells (135).

Although piRNA regulation of human CSCs remains unclear, the upregulation of *piR-823* was identified in tested luminal breast cancer cells, resulting as a potential oncogenic regulator of cell proliferation and colony formation. Its upregulation increases the expression of DNMTs, promoting DNA methylation of APC gene, activating Wnt signaling and inducing cancer cell stemness; this contributes to tumorigenesis, thus representing a promising target for treatment (136, 137).

On the other hand, piRNAs can also have a tumor suppressive effect: *piR-36712* inhibits *SEPW1* expression, consequently increasing wild-type P53, P21, and E-cadherin levels; at the same time, it decreases SLUG levels, with a significant reduction in proliferation, migration, and invasion. Interestingly, *piRNA-36712* has also a synergistic anticancer effect combined with chemotherapeutic agents (Paclitaxel and Doxorubicin) for BC cells (138).

Noteworthy, *piR-2158* contributes to the inhibition of mammary gland tumorigenesis *via* regulating cancer stem cells and tumor angiogenesis (139, 140), it competes with FOSL1 resulting in the inhibition of IL-11 (141, 142), a key regulator of cancer cell stemness and tumoral growth (143). A recent study by Wu and colleagues detected a novel PIWI-interacting RNA that could have a protective role in BC (144): *piR-YBX1*, whose overexpression significantly inhibited the proliferation, migration, and invasion ability of TNBC cells both *in vivo* and *in vitro*. When upregulated, *piR-YBX1* binds *YBX1* mRNA leading to its degradation and markedly lowering its expression at both transcript and protein levels (145–147). YBX1 has a well-known oncogenic activity, and some ncRNAs can interact with it influencing directly cancer progression (148–150). There are other possible oncogenes degraded by *piR-YBX1*, but more evidence is required to confirm this thesis.

Interestingly, YBX1 can influence TNBC cancer development by regulating the MAPK signaling pathway *via* binding RAF1; this mechanism has a pivotal role in reverting the effects of *piR-YBX1* overexpression. It becomes important then to state that the effect of *agopiR-YBX1* on the inhibition of distant metastasis is still not proven (144). There are many interrogatives regarding piRNA biology and mechanisms, especially in the modulation of various diseases, but recent innovations in RNA sequencing methods, such as piRNA single-cell RNA-seq and spatial RNA-seq are promising tools for a rapid development of the field of piRNAs in tumors. In fact, these ncRNAs could be potential biomarkers in cancer diagnosis and treatment, but simultaneously, multiple independent, large-scale and prospective cohorts are needed to validate their effectiveness (151).

### 5.3 Small nuclear RNAs

Small nuclear RNAs (snRNAs) are small non-coding RNAs located in the Cajal bodies (CBs) and splicing speckles in the nucleus (152); they are present in all eukaryotic cells and account approximately for about 1% of total mammalian cellular RNA (153). These highly abundant nuclear RNAs form the core of ribonucleoprotein particles, called snRNPs, which function by splicing introns from primary genomic transcripts and play important roles in gene expression (154). Each snRNP comprises post-transcriptionally modified uridylic acid-rich small nuclear RNA and a cortege of associated proteins (155, 156). Based on their function and intra-nuclear localization, mammalian snRNPs can be classified into three major classes: major and minor spliceosomal snRNPs (respectively, *U1*, *U2*, *U4*, *U5*, *U6* and *U11*, *U12*, *U4atac* and *U6atac*) that function in the removal of pre-mRNA introns and are predominantly nucleoplasmic; and a third group composed by the small Cajal body RNPs (scaRNPs), that accumulate in CBs and direct the site-specific 2′-O-ribose methylation and pseudo-uridylation of the RNA polymerase (Pol)-II-transcribed spliceosomal snRNAs. Recent data suggest that they could fill additional roles in gene expression regulation: *U1* and *U2* have been implicated in transcriptional regulation, with *U1* enhancing the first phosphodiester bond formation during the beginning of transcription and with *U2* interacting with a component of the pre-initiation complex Transcription Factor II H (TFIIH) (157, 158). Furthermore, a potential RNA degradation can be caused by the polyadenylation inhibition derived from the *U1* bond to the 5′ splice site-like sequence in the 3′UTR of some mRNAs.

Some studies have shown that the abundance of snRNA can be regulated under some cell stress conditions (159); *U6atac* relies on both RNA polymerases II and III and its levels rise as a stress-respond increased by activating the p38MAPK pathway. The kinase stabilizes *U6atac*, promoting the expression of numerous minor intron-containing genes that are otherwise repressed consequently to a low *U6atac* availability. This mechanism can also influence the expression of key genes (as *PTEN)* and modulates cytokine production (160).

In cancer, aberrant mRNA splicing is frequent. Nevertheless, there has been minimal analysis of snRNAs as “basal factors” required for catalyzing the process. *U1* is one of the most abundant ncRNAs in human cells and plays an important role in splicing pre-mRNAs, aberrancies in this process are considered a primary cause of human disease (161). Dvinge and colleagues depicted that, although *U1*, *U2*, *U4*, *U5*, and *U6* snRNA are present in equal stoichiometry within the spliceosome, their relative levels vary during development across tissues and across cancer samples, especially in the context of BCs. This suggests that snRNA levels play important roles in establishing tissue-specific and developmental stage-specific splicing programs (162). In the same manuscript, scientists pointed out how snRNAs dysregulation can shape the global transcriptome of breast cancer and contribute to tumorigenesis itself: *U1* and *U5A* were abundantly found in HER2+ BC subtype, whereas the two clusters of triple-negative analyzed samples showed a higher relative presence of *U6* and comparatively low levels of *U2* and *U5A*. Undoubtedly, further work is required to determine their effective contribution to the definition and/or regulation of each subtype (162). A recent work by Caggiano and colleagues evidenced that the inhibition of U2 snRNP induces persistent DNA damage in triple-negative breast cancer cells and organoids; this inhibition downregulates genes involved in DNA damage response (DDR), whose structure is characterized by numerous small exons and that are expressed at high levels in TNBC (163). DDR genes comprising many exon-intron junctions are probably more likely to be affected by splicing inhibition because of the numerous splicing reactions required to process them. For instance, *BRCA1/2* and *ATRIP* are among the most affected genes. This window of vulnerability in TNBC cells could be exploited therapeutically (163).

*U1* snRNA exerts a significant impact also on migration and invasion in breast cancer cell lines, activating proto-oncogenes and downregulating ORF-disruptive splicing changes in tumor suppressors (like ATM). U1 inhibition results in premature transcription termination and mRNA shortening; conversely, *U1* over-expression negates these effects and significantly decreases cell line ability to migrate and spread (164), presenting a suitable target for inhibiting BC invasion. *U1* can also silence the polyadenylation signals (PAS) activity, leading to shortened mRNA 3-UTR regions and therefore shortened mRNA isoforms, typical of certain cell types but also present in various cancers, including BC (165).

Thanks to their location in the nucleus, snRNAs can be detected through liquid biopsy and potentially be used for early non-invasive cancer detection (Table 5). An example is represented by the persistence of elevated levels of *U6* in the plasma of ER+ and ER-breast cancer patients, both active and inactive, but not in healthy cases; this evidence hence indicates an increased polymerase III activity in breast tumors, regardless of the disease progression (166, 167).

**TABLE 5:** Small nuclear RNAs scheme in Breast Cancer types.

Small nuclear RNAs (snRNAs)		
Regulation	piRNAs	BC type
Biomarkers	(plasma) *U6* (166–168)	HR +
	*U1*; *U5A* (158, 162, 164, 165, 169, 170)	HER2 +
	*U6* (166–168)	TNBC

### 5.4 Small nucleolar RNAs

Small nucleolar RNAs (snoRNAs) are non-coding RNAs ranging from 60 to 300 bp, primarily located in the nucleoli of eukaryotic cells, and generally derived from intronic sequences. They are categorized into C/D box snoRNAs, H/ACA box snoRNAs and scaRNAs (small Cajal RNAs). The first two modify ribosomal RNA (rRNA) through 2′-O-methylation and pseudouridylation, respectively, while scaRNAs localize in the Cajal bodies (171). These snoRNAs associate with specific proteins to form RNPs. C/D snoRNAs possess conserved C and D motifs (located at the 5′ and 3′, respectively), forming “kink-turn” structures recognized by binding proteins (172). H/ACA snoRNAs include H and ACA motifs and feature “pseudouridylation pockets” targeting uridines in rRNA (173). Some snoRNAs, lacking an apparent complementarity with rRNAs at known modified positions, are called “orphan snoRNAs,” and may play broader roles beyond canonical rRNA modifications (174).

In recent years, there has been increasing interest, and several studies have confirmed that snoRNAs are involved in processes like alternative splicing, ac4C modifications, and even miRNA-like activity, positioning snoRNAs as regulators of cellular function (174–178). A 2016 work by Krishnan and colleagues reported over 40 snoRNAs differentially expressed in breast cancer tissue, of which 13 can have prognostic significance (179). Besides, functional studies suggest that snoRNAs can be up- or downregulated in BC, acting as oncogenes or tumor suppressors (180). For example, the snoRNA host gene *ZFAS1* is downregulated in breast cancer, and it might control cellular homeostasis, proliferation and differentiation (181). Elevated snoRNAs and fibrillarin (FBL, an enzymatic snoRNP) expression has been linked to impaired p53 activation and increased tumorigenicity (182), while snoRNA *U50*, usually downregulated in prostate and breast cancer, has a significant correlation with tumor grade (183). It mediates the methylation of *C2848* in 28 S rRNA, acting as a potential tumor-suppressor gene (184, 185).

Other small nucleolar RNAs, such as *SNORD50A* and *SNORD50B* (*SNORD50A/B*), negatively regulate KRAS oncoproteins and modulate p53 signaling through GMPS interaction (186, 187). Conversely, small nucleolar RNAs can also be involved in BC development and metastasis, *U3* and *U8* (upregulated in BC tissues) are essential for pre-rRNA processing reactions, leading to the synthesis of the small and large ribosomal subunits. Their depletion triggers p53-mediated anti-tumor stress responses. Tumors derived from *U3*-knockdown cells displayed markedly lower metabolic volume and activity than tumors derived from aggressive control cancer cells; this indicates distinctive tumor growth properties that may reflect non-conventional regulatory functions of *U3* in mRNA metabolism (188). The overexpression of small nucleolar RNA host genes (*SNHGs*) like *SNHG1* and *SNGH3* influences proliferation, migration, and EMT through regulation of miRNAs (like *miR-186-5p*, *miR-154-3p*, *miR-330-5p* and *miR-384*) and key oncogenic pathways (such as Notch signaling) (189–193).

*SNORA73A*, *SNORA73B*, and *SNORA74A* are also bound to PARP-1 to activate its catalytic activity and mediate ADPRylation of DDX21, promoting cell proliferation in BC (194). In addition, *SNORA71A* also promotes the binding of G3BP1-ROCK2 and increases the expression of *ROCK2*, promoting the EMT process (195).

SnoRNA-derived fragments (sno-miRNAs or sdRNAs) such as *sdRNA-93* and *sno-miR-28*, exhibit microRNA-like behavior, promoting cancer cell invasion and affecting genes like *Pipox* and *TAF9B*, which stabilizes p53 in physiological conditions. A brief explanation of this process is that the interaction between p53, *NHG1*, *sno-miR-28*, and *TAF9B* results in a signaling cascade, which significantly affects p53 and modifies its downstream gene network (196–198).

All these various implications highlight the potential diagnostic value of these non-coding RNAs (Table 6). Multiple studies have highlighted that snoRNAs are also detectable in body fluids like blood, plasma and urine, suggesting their utility as non-invasive cancer biomarkers. For instance, a very recent study identified four snoRNAs–*SNORD16*, *SNORA73B*, *SCARNA4*, and *SNORD49B*–that are significantly increased in the plasma of breast cancer patients, especially in early-stage patients, representing an interesting diagnostic potential. Nevertheless, how snoRNAs facilitate BC cells acquiring cancer hallmarks and contribute to therapeutic sensitivity or resistance is unknown. Besides, the cell signaling pathways, molecular mechanisms, and their regulation are unclear and require detailed investigation (199).

**TABLE 6:** Small nucleolar RNAs scheme in breast cancer types.

Small nucleolar RNAs (snoRNAs)		
Regulation	snoRNAs	BC type
Breast cancer proliferation/tumorigenesis	FBL (snoRNP) (182); *SNORD22*; *SNORD25*; *SNORD26*; *SNORD27*; *SNORD28*; *SNORD29*; *SNORD30*; *SNORD31* (189–193).; *SNORA73A*; *SNORA73B*; *SNORA74A* (194, 195); *sno-miR-28* (196)	Not defined
Breast cancer metastasis	*U3*; *U8* (188)	Not defined
Tumor suppression	*U50* (183, 184); *SNORD50A/B* (186, 187)	wild type p53
Biomarkers	*SNORD16*; *SNORA73B*; *SCARNA4*; *SNORD49B* (199)	not defined

## 6 Conclusion

As thoroughly discussed, non-coding RNAs represent a vast resource in the comprehension and treatment of breast cancer; they act at different levels and with different mechanisms both in the development and in the inhibition of these tumors. All the direct and indirect correlations described in this manuscript suggest non-coding RNAs as perfect candidates as biomarkers for diagnosing tumors, judging patient prognosis, and predicting disease progression. Moreover, multiple studies have proved that many of these ncRNAs are stably expressed in BC patients’ blood, plasma, urine, and other body fluids even at early stages, providing evidence for a novel class of non-invasive biomarkers for BC.

Among them, an emerging and promising diagnostic tool is represented by circRNAs. Their unique structure, as mentioned above, provides them with stability and specificity, making them interesting candidates for therapeutic strategies. Furthermore, many studies show circRNAs ability to regulate BC cell sensibility to treatment, predicting responsiveness to chemo-, radio-, immuno-, and hormone-therapies, highlighting their potential to overcome the resistance issue. All these various implications highlight the potential diagnostic value of these non-coding RNAs.

Furthermore, with the continuous discovery of ncRNAs structural information and regulatory functions, small molecule inhibitors against ncRNAs have been developed with broad prospects for clinical diagnosis and treatment of tumors. RNA interference (RNAi) can be harnessed to inhibit the expression of cognate mRNA by exogenous RNA-based molecules that can be synthetically designed against any target RNA. The new anti-tumor drugs against ncRNAs have become a new promising trend in cancer treatment. At present, the research of new molecules targeting ncRNAs has made some progress.

a. small (or short) interfering RNAs, siRNAs, which target transcripts via RNA-induced silencing complex (RISC), downregulating mRNA levels;
b. miRNAs sponges, molecules designed as decoys that specifically target microRNA seed families;
c. antisense oligonucleotides (e.g., ASOs or LNA Gapmers) that hybridize with their target RNA, blocking the formation of its secondary structure and mediating degradation by the RNAse-H;
d. aptamers, nucleic acid-based structures that act similarly to antibodies and interfere with the RNA tertiary structure, through which they associate with their interactors;
e. CRISPR-Cas9 technology, which may be exploited for targeted repression via guide RNAs but can also restore expression of dormant ncRNAs with tumor suppressor properties;
f. indirect modulators of lncRNAs are also a new direction in drug development.

There are many examples of RNAi, such as siRNAs, that can target all kinds of proteins, including traditionally undruggable proteins, and there is also evidence that these molecules could be superior to antibodies or small molecule inhibitors when inhibiting the same pathway, demonstrating a high therapeutic potential (200).

Although ncRNAs are opening a new door for clinical diagnosis and treatment of breast cancer and these nucleic acid-based approaches have great potential for clinical application, their limitations should be considered, and further investigations are needed to.

• increase their stability,
• avoid rapid degradation,
• evaluate off-target effects due to possible sequence pairing,
• implement an efficient delivery system for tissue recognition and intercellular localization
• overcame immune barriers.

In this perspective, several strategies are under investigation to efficiently deliver these molecules against BC-related ncRNA targets, such as synthetic ionizable lipids (LNP), including ASOs targeting *MALAT1* and *HOTAIR* lncRNAs (201) or *ZIF-90* (202) nanoparticles enveloping dual antisense oligonucleotide targeting *miR-21/miR-155* to treat TNBC and inhibit metastasis.

Undoubtedly, research improvements in this field will provide more action strategies in the understanding of BC cancer biology, in its correct diagnosis, and in the development of personalized, targeted therapies that may also be helpful in the more aggressive forms of the disease, opening new avenues for precision medicine in a heterogeneous disease such as breast cancer.
